# YCRD: Yeast Combinatorial Regulation Database

**DOI:** 10.1371/journal.pone.0159213

**Published:** 2016-07-08

**Authors:** Wei-Sheng Wu, Yen-Chen Hsieh, Fu-Jou Lai

**Affiliations:** Department of Electrical Engineering, National Cheng Kung University, Tainan, Taiwan; University College London, UNITED KINGDOM

## Abstract

In eukaryotes, the precise transcriptional control of gene expression is typically achieved through combinatorial regulation using cooperative transcription factors (TFs). Therefore, a database which provides regulatory associations between cooperative TFs and their target genes is helpful for biologists to study the molecular mechanisms of transcriptional regulation of gene expression. Because there is no such kind of databases in the public domain, this prompts us to construct a database, called Yeast Combinatorial Regulation Database (YCRD), which deposits 434,197 regulatory associations between 2535 cooperative TF pairs and 6243 genes. The comprehensive collection of more than 2500 cooperative TF pairs was retrieved from 17 existing algorithms in the literature. The target genes of a cooperative TF pair (e.g. TF1-TF2) are defined as the common target genes of TF1 and TF2, where a TF’s experimentally validated target genes were downloaded from YEASTRACT database. In YCRD, users can (i) search the target genes of a cooperative TF pair of interest, (ii) search the cooperative TF pairs which regulate a gene of interest and (iii) identify important cooperative TF pairs which regulate a given set of genes. We believe that YCRD will be a valuable resource for yeast biologists to study combinatorial regulation of gene expression. YCRD is available at http://cosbi.ee.ncku.edu.tw/YCRD/ or http://cosbi2.ee.ncku.edu.tw/YCRD/.

## Introduction

Transcriptional regulation is one of the major mechanisms for cells to control the timing, location, and amount of gene expression. The precise transcriptional control of gene expression is typically achieved through combinatorial regulation using cooperative transcription factors (TFs) [1–3]. Therefore, to understand how a gene of interest is transcriptionally regulated, it is crucial to know the cooperative TFs which function together to regulate the gene.

YEASTRACT [4] database provides up-to-date information on experimentally validated regulatory associations between a TF and its target genes. By querying YEASTRACT, users can know the TFs which regulate a specific gene. However, one key information is missing. Users cannot know whether these TFs function cooperatively or independently in regulating the expression of the specific gene. Therefore, it would be helpful to have a database which provides regulatory associations between cooperative TFs and their target genes. Because there is no such kind of databases in the public domain, this prompts us to construct the Yeast Combinatorial Regulation Database (YCRD).

In YCRD, we collected more than 2500 cooperative TF pairs predicted by 17 existing algorithms in the literature [5–21]. As far as we know, this is the most comprehensive collection of predicted cooperative TF pairs in yeast (see Table 1 for details). Moreover, we retrieved a TF’s experimentally validated target genes from YEASTRACT database [4]. Then for each cooperative TF pair (e.g. TF1-TF2), we define its target genes as the common target genes of TF1 and TF2. Therefore, the regulatory associations between cooperative TF pairs and their target genes are of biological significance because they are experimentally validated. In YCRD, users can search the target genes of a cooperative TF pair of interest validated by different types of experimental evidence (TF binding evidence or/and TF regulation evidence). Users can also search the cooperative TF pairs which regulate a gene of interest validated by different types of experimental evidence. Moreover, for a given set of genes, YCRD provides a tool for identifying important cooperative TF pairs which regulate these genes. We believe that YCRD will be a valuable resource for yeast biologists to study combinatorial regulation of gene expression. YCRD is freely available at http://cosbi.ee.ncku.edu.tw/YCRD/ or http://cosbi2.ee.ncku.edu.tw/YCRD/.

**Table 1:** The ratio of the number of predicted cooperative TF pairs (PCTFPs) to the number of TF pairs under study for each of the 17 existing algorithms in the literature.

Existing algorithms	# of PCTFPs / # of TF pairs under study	Existing algorithms	# of PCTFPs / # of TF pairs under study
Banerjee and Zhang (2003) [5]	$31/\left(\begin{array}{c} 113\\ 2 \end{array}\right)$	Elati et al. (2007) [14]	$20/\left(\begin{array}{c} 466\\ 2 \end{array}\right)$
Harbison et al. (2004) [6]	$94/\left(\begin{array}{c} 203\\ 2 \end{array}\right)$	Datta and Zhao (2008) [15]	$25/\left(\begin{array}{c} 203\\ 2 \end{array}\right)$
Nagamine et al. (2005) [7]	$24/\left(\begin{array}{c} 113\\ 2 \end{array}\right)$	Chuang et al. (2009) [16]	$13/\left(\begin{array}{c} 203\\ 2 \end{array}\right)$
Tsai et al. (2005) [8]	$18/\left(\begin{array}{c} 203\\ 2 \end{array}\right)$	Wang et al. (2009) [17]	$159/\left(\begin{array}{c} 184\\ 2 \end{array}\right)$
Balaji et al. (2006) [9]	$3459/\left(\begin{array}{c} 157\\ 2 \end{array}\right)$	Yang et al. (2010) [18]	$186/\left(\begin{array}{c} 178\\ 2 \end{array}\right)$
Chang et al. (2006) [10]	$55/\left(\begin{array}{c} 203\\ 2 \end{array}\right)$	Chen et al. (2012) [19]	$221/\left(\begin{array}{c} 203\\ 2 \end{array}\right)$
He et al. (2006) [11]	$30/\left(\begin{array}{c} 113\\ 2 \end{array}\right)$	Lai et al. (2014) [20]	$27/\left(\begin{array}{c} 186\\ 2 \end{array}\right)$
Wang (2006) [12]	$14/\left(\begin{array}{c} 9\\ 2 \end{array}\right)$	Wu and Lai (2015) [21]	$50/\left(\begin{array}{c} 151\\ 2 \end{array}\right)$
Yu et al. (2006) [13]	$300/\left(\begin{array}{c} 113\\ 2 \end{array}\right)$		

$\left(\begin{array}{c} N\\ 2 \end{array}\right)=\frac{N!}{2!(N-2)!}$ means all possible pairs formed by *N* TFs.

## Construction and Contents

### The comprehensive collection of predicted cooperative TF pairs from 17 existing algorithms in the literature

Many existing algorithms have been developed to predict cooperative TF pairs in yeast. Each algorithm utilized distinct biological rationales, reported its own set of predicted cooperative TF pairs (PCTFPs) and performed differently under different evaluation indices [22,23]. We comprehensively collected 3755 distinct cooperative TF pairs from 17 existing algorithms (see Table 1 for details). Lai et al. [22] had good background information on 14 existing algorithms. Here we briefly introduce the other three algorithms. Baljai et al. [9] regarded a TF pair as a PCTFP if the observed number of shared target genes is higher than random expectation. Lai et al. [20] regarded a TF pair as a PCTFP if (i) the two TFs have a significantly higher number of common target genes than random expectation and (ii) their binding sites tend to be co-depleted of nucleosomes. Wu and Lai [21] regarded a TF pair as a PCTFP if the overlap of the targets of these two TFs is higher than random expectation. As far as we know, our collection of 3755 distinct cooperative TF pairs is the most comprehensive collection of predicted cooperative TF pairs in the literature. Note that we collected predicted rather than experimentally verified cooperative TF pairs. This is because the number of experimentally verified cooperative TF pairs in the literature is too small to construct a useful database of combinatorial regulation of gene expression.

Among the 3755 collected PCTFPs, 1133 PCTFPs which contained non-TF names were removed. A protein name is regarded as a TF name only if it is annotated as a TF (activator/repressor) or a transcription co-factor in the regulation page of SGD [24]. After this curation, we obtained 2622 PCTFPs among 143 TFs. We then removed 87 PCTFPs which have no target genes (see the next subsection for how to define the target genes of a PCTFP). Finally, 2535 cooperative TF pairs were used in this database. In order to help users judge the biological plausibility of a PCTFP, we provide three types of validation. The first type, called Algorithm Evidence, tells users the ratio of the number of existing algorithms which predict this PCTFP (e.g. TF1-TF2) to the number of existing algorithms which study this TF pair (e.g. TF1-TF2). The higher the ratio is, the higher the confidence of this PCTFP is. The second/third type, called Physical/Genetic Evidence, tells users the number of publications which experimentally show that this PCTFP has physical/genetic interactions. Having physical or genetic interactions strengthens the confidence of the biological plausibility of this PCTFP. The physical and genetic interaction data were retrieved from BioGRID database [25].

### Defining the target genes of a cooperative TF pair

YEASTRACT [4] database uses three types of experimental evidence (TFB, TFR and TFB&TFR) to define a TF’s experimentally validated target genes. TFB (i.e. TF binding) means the experimental evidence (from band-shift, foot-printing or ChIP assay) showing that a TF binds to the promoters of its target genes. TFR (i.e. TF regulation) means the experimental evidence (from detailed gene by gene analysis or genome-wide expression analysis) showing that a TF perturbation (knockout or over-expression) causes a significant change in the expression of its target genes. TFB&TFR means both TFB and TFR evidence exist to support the regulatory associations between a TF and its target genes.

In YCRD, the target genes of a cooperative TF pair (e.g. TF1-TF2) are defined as the common target genes of TF1 and TF2, where a TF’s experimentally validated target genes were retrieved from the YEASTRACT database [4]. For example, we say that X is a target gene of the cooperative TF pair (TF1-TF2) validated by TFB evidence if both the regulatory association between TF1 and X and the association between TF2 and X are validated by TFB evidence. However, in the above definition, the TFB evidences of TF1-X and TF2-X may not be in the same biological condition. If we further require that the TFB evidences of TF1-X and TF2-X must be in the same biological condition, then, on average, the number of the target genes of a cooperative TF pair reduces to 53% (33346/63418) under this stringent definition (see S1 File for details). Since many biological conditions have not been tested for TFB evidence in the literature, it may be too conservative to use the stringent way to define the target genes of a cooperative TF pair. Therefore, we use the less stringent way to define the target genes of a cooperative TF pair.

After collecting cooperative TF pairs and defining the target genes of each cooperative TF pair, a web interface is then constructed for users to query the regulatory associations (validated by TFB, TFR or TFB&TFR) between cooperative TF pairs and their target genes. The detailed statistics of YCRD could be found in Table 2.

**Table 2:** The detailed statistics of YCRD.

	# of regulatory associations	# of cooperative TF pairs which can be assigned at least one target gene	# of genes which can be assigned at least one cooperative TF pair
**Regulatory associations validated by TFB evidence**	63,418	2,441	4,119
**Regulatory associations validated by TFR evidence**	364,360	2,519	6,165
**Regulatory associations validated by TFB&TFR evidence**	6,419	1,013	1,380

### Identifying important cooperative TF pairs which regulate a given set of genes

When researchers have a set of genes (e.g. upregulated genes under a specific biological condition), they probably want to know the cooperative TF pairs which play important roles in regulating these genes. To meet this need, YCRD provides a tool for identifying important cooperative TF pairs which regulate a given set of genes. In YCRD, a cooperative TF pair is regarded as an important regulator if its target genes are enriched in the given set of genes. The hypergeometric distribution is used to test the statistical significance of enrichment [26]. The procedure for checking whether a cooperative TF pair is an important regulator for a given set of genes is as follows. Let *S* be the set of target genes of a cooperative TF pair of interest, *G* be the given set of genes, *T* = *S* ∩ *G* be the set of the cooperative TF pair’s target genes which are also in the given set of genes, and *F* be the set of all genes in the yeast genome. Then the *p*-value for rejecting the null hypothesis (H_0_: the cooperative TF pair’s target genes are not enriched in the given set of genes) is calculated as

$$p_{value}=P(x\geq |T|)=\sum_{x\geq |T|}\frac{\left(\begin{array}{c} \left|S\right|\\ x \end{array})(\begin{array}{c} \left|F|-|S\right|\\ |G|-x \end{array}\right)}{\left(\begin{array}{c} \left|F\right|\\ \left|G\right| \end{array}\right)}$$ (1)

where |*G*| means the number of genes in set *G*. This *p*-value is then corrected by the Bonferroni correction to represent the true alpha level in the multiple hypotheses testing. A cooperative TF pair of interest is called an important regulator for the given set of genes if the Bonferroni-corrected *p*-value is less than the threshold determined by users. We also allow users to specify the false discovery rate (FDR) when identifying important cooperative TF pairs which regulate a given set of genes.

## Utility and Discussion

### Database interface

**Fig 1:**
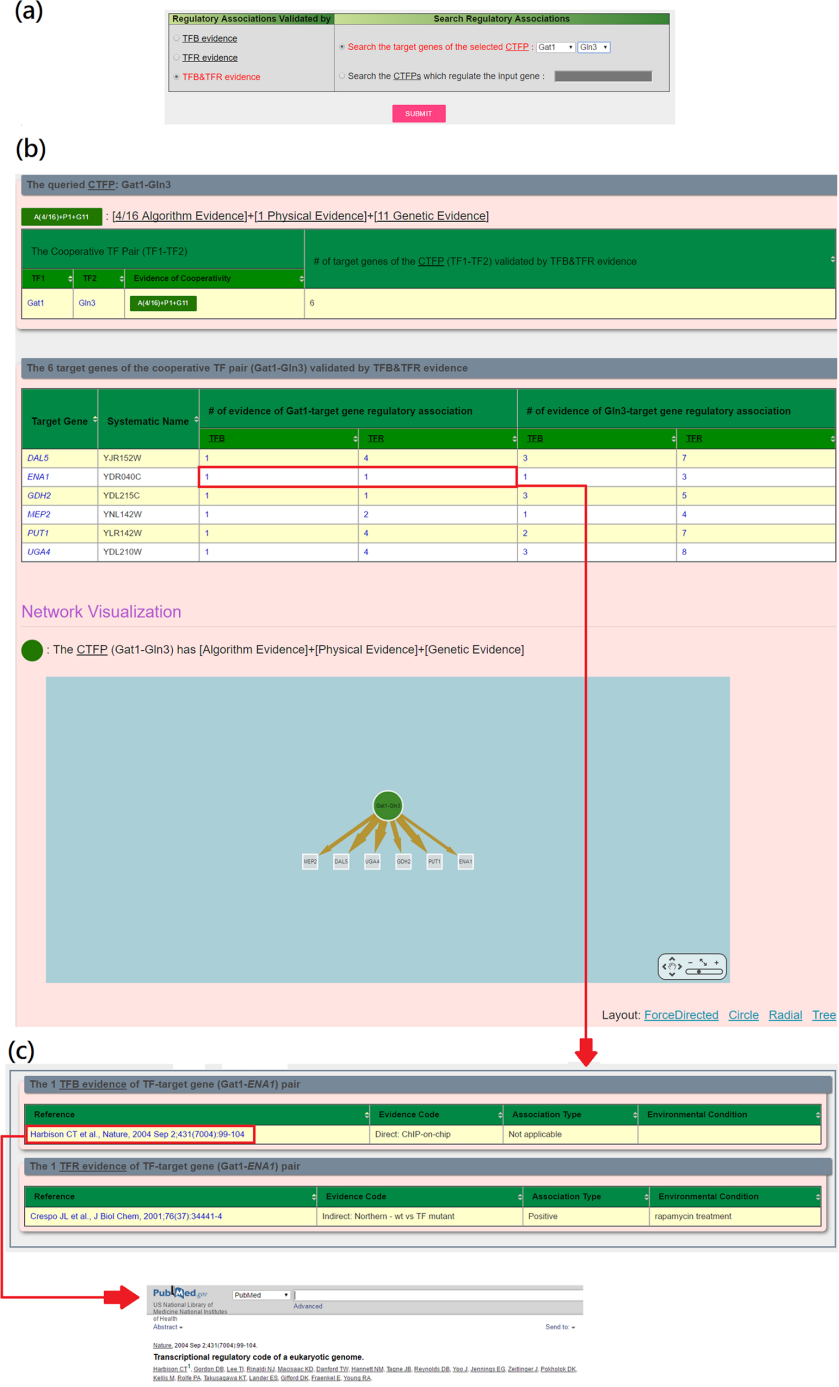
Search the target genes of a cooperative TF pair of interest. (a) The input page. (b) The result page. (c) The publications of the experimental evidence of the regulatory associations.

**Fig 2:**
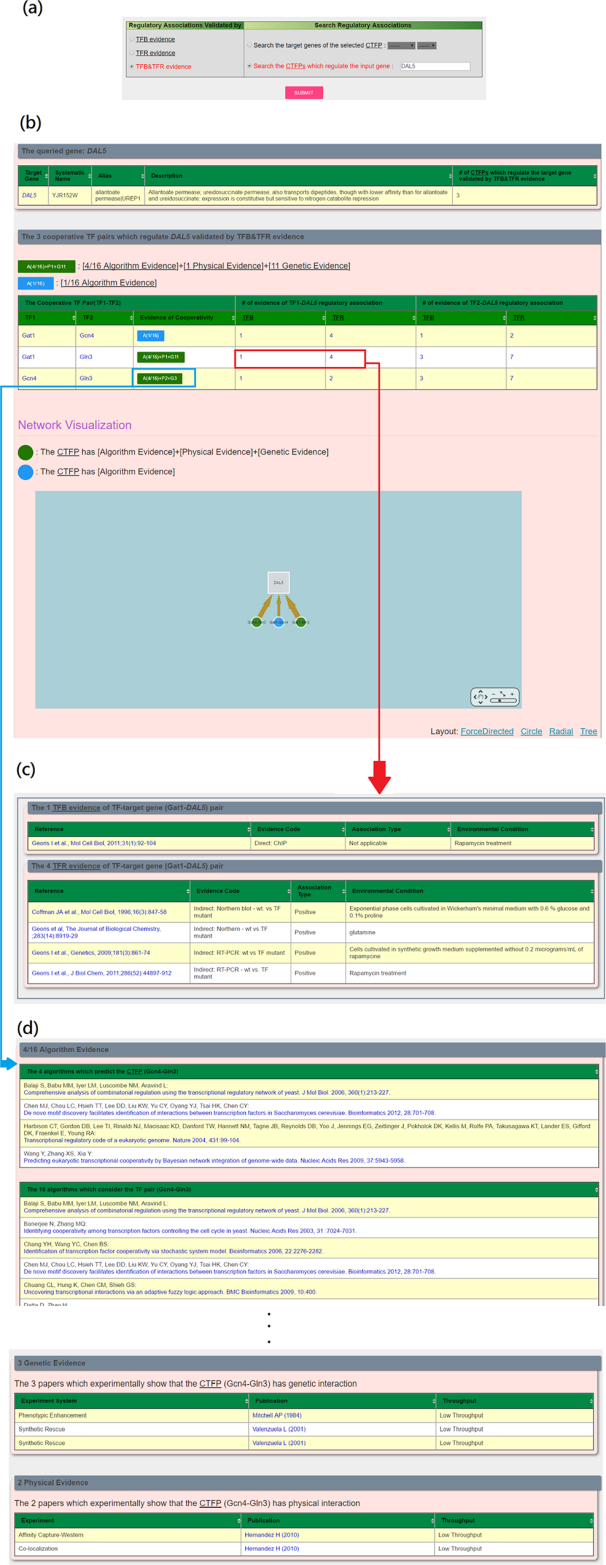
Search the cooperative TF pairs which regulate a gene of interest. (a) The input page. (b) The result page. (c) The publications of the experimental evidence of the regulatory associations. (d) Three types of validation (Algorithm Evidence, Physical Evidence and Genetic Evidence) for each cooperative TF pair.

YCRD provides a search mode and a browse mode. In the search mode, users have two possible ways to search YCRD. First, users can (i) select the experimental evidence (TFB, TFR or TFB&TFR) of the regulatory associations and (ii) select a cooperative TF pair of interest (see Fig 1A). Then YCRD returns the target genes of the selected cooperative TF pair shown in a table and a figure (see Fig 1B). The publications of the experimental evidence of the regulatory associations are also provided (see Fig 1C). Second, users can (i) select the experimental evidence (TFB, TFR or TFB&TFR) and (ii) type in the name of a gene of interest (see Fig 2A). Then YCRD returns the cooperative TF pairs which regulate the input gene shown in a table and a figure (see Fig 2B). The publications of the experimental evidence are also provided (see Fig 2C). Moreover, three types of validation (Algorithm Evidence, Physical Interaction Evidence and Genetic Interaction Evidence) for each cooperative TF pair are given (see Fig 2D).

**Fig 3:**
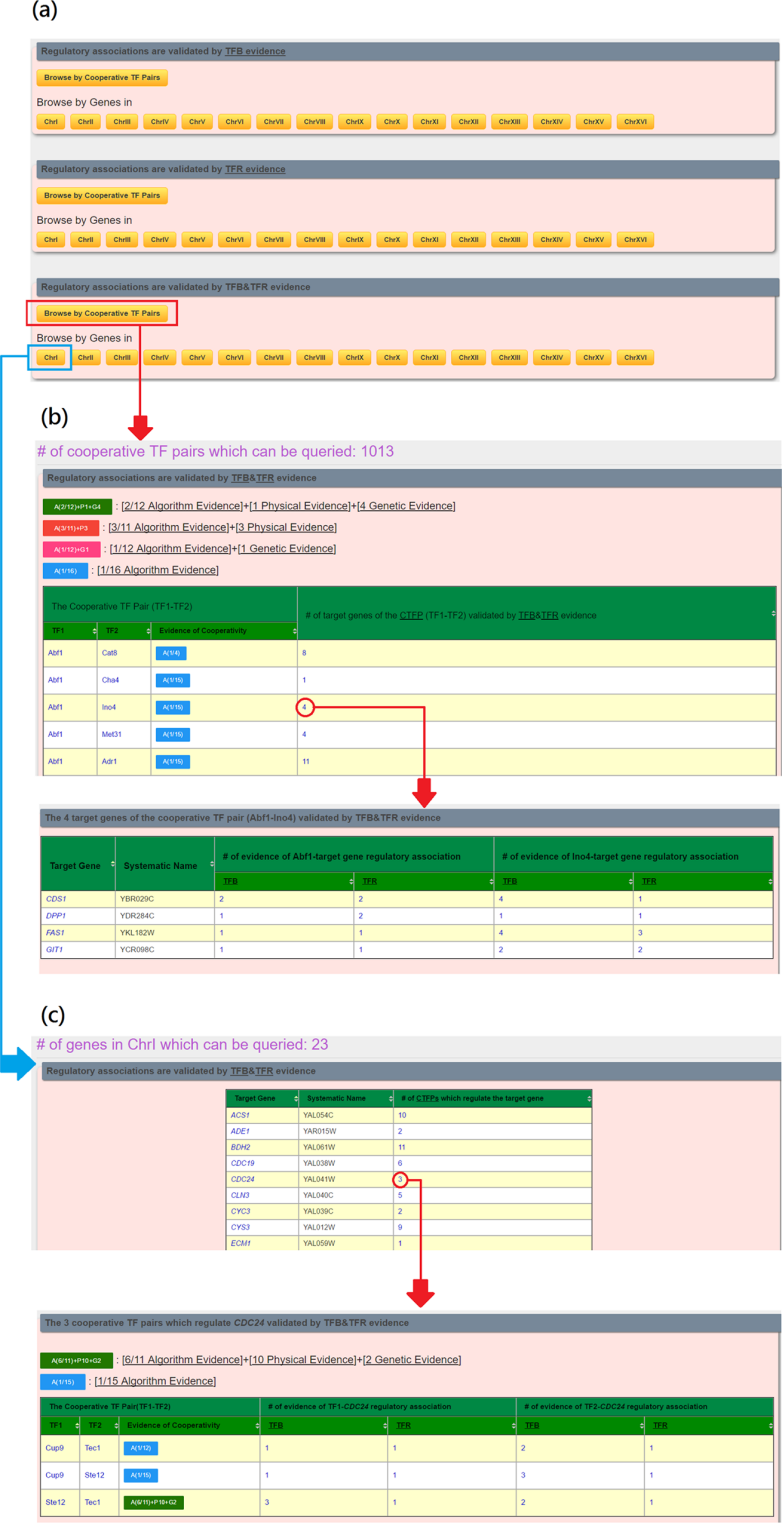
The browse mode. (a) The browse page. (b) The result page of browsing YCRD by the name of a cooperative TF pair. (c) The result page of browsing YCRD by a gene name.

In the browse mode, users have two possible ways to browse the regulatory associations (validated by TFB, TFR or TFB&TFR) between cooperative TF pairs and their target genes (see Fig 3A). First, when users browse YCRD by the name of a cooperative TF pair, users will be given the target genes of this cooperative TF pair (see Fig 3B). Second, when users browse YCRD by a gene name, users will be given the cooperative TF pairs that regulate this gene (see Fig 3C).

**Fig 4:**
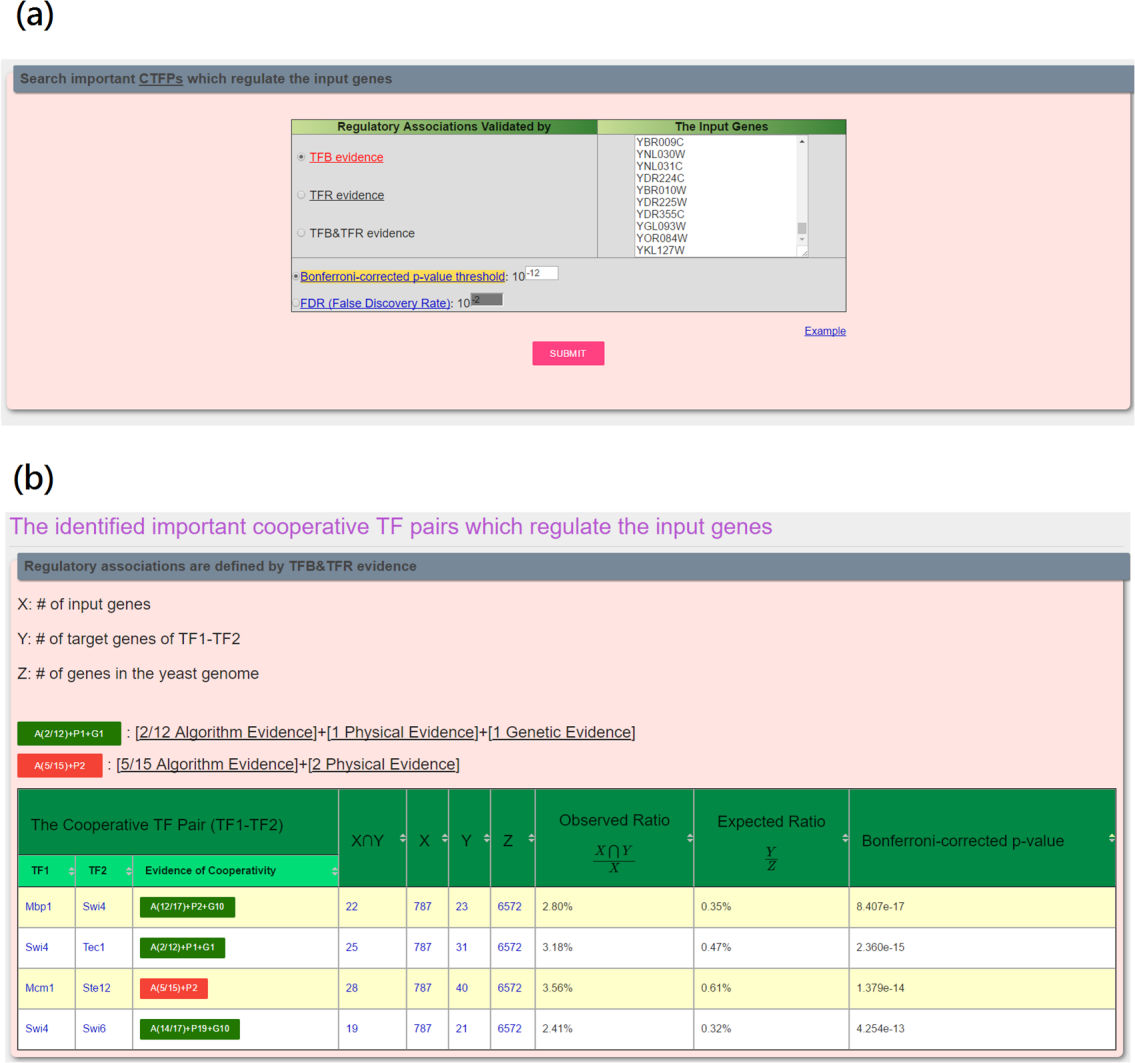
A tool for identifying important cooperative TF pairs which regulate a given set of genes. (a) The input page. (b) The result page.

YCRD also provides a tool for identifying important cooperative TF pairs which regulate a given set of genes. To use this tool, users have to (i) input a set of genes of interest, (ii) choose the experimental evidence (TFB, TFR or TFB&TFR) of the regulatory associations, and (iii) set the threshold of the Bonferroni-corrected p-value, where p-value is calculated using Eq (1) (see Fig 4A). YCRD then returns the important cooperative TF pairs which regulate the set of genes of interest (see Fig 4B).

### Three case studies

We use three case studies to show that YCRD is likely to return biologically meaningful results. In the first case study, we would like to search the target genes of the CTFP (Gat1-Gln3) with the regulatory associations validated by TFB&TFR. After submission, YCRD returns six target genes (*DAL5*, *ENA1*, *GDH2*, *MEP2*, *PUT1*, *UGA4*) (see Fig 1B). TFB evidence shows that each target gene’s promoter is bound by Gat1 and Gln3. TFR evidence shows that the expression of each target gene is significantly changed when perturbing the expression of the gene encoding Gat1 or Gln3 (see Fig 1C). We validate the biological relevance of the six identified target genes as follows. First, it is known that Gat1 and Gln3 are two TFs which cooperatively activate a set of genes involved in nitrogen catabolite pathways [27]. Strikingly, four (*DAL5*, *GDH2*, *PUT1* and *UGA4*) of the six identified target genes are known genes involved in nitrogen catabolite pathways [27]. Second, the expression of *ENA1*, a gene encoding a lithium and sodium ion transporter essential for salt tolerance in yeast, is known to be regulated by the transcriptional complex Gat1-Gln3 [28]. Third, Gat1 and Gln3 are known to cooperatively regulate *MEP2*, a gene encoding an ammonium permease, whose expression is induced under limiting nitrogen conditions [29].

In the second case study, we would like to search the CTFPs which regulate the gene *DAL5* with the regulatory associations validated by TFB&TFR. After submission, YCRD returns three CTFPs (Gat1-Gln3, Gcn4-Gln3 and Gat1-Gcn4) (see Fig 2B). TFB evidence shows that the promoter of *DAL5* is bound by Gat1, Gcn4 and Gln3. TFR evidence shows that the expression of *DAL5* is significantly changed when perturbing the expression of the gene encoding Gat1, Gcn4 or Gln3 (see Fig 2C). We validate the biological relevance of the three identified CTFPs as follows. First, it is known that *DAL5*, a gene encoding an allantoate permease, is involved in nitrogen catabolite pathways [27]. The identified CTFP Gat1-Gln3 is known to form a transcriptional complex to regulate *DAL5* in response to nitrogen limitation [27]. Second, the identified CTFP Gcn4-Gln3 is known to form a transcriptional complex to regulate *DAL5* when cells are grown under nitrogen derepressive conditions and amino acid deprivation [30]. However, the identified CTFP Gat1-Gcn4 has no physical or genetic interaction. We suspect that the cooperativity between Gat1 and Gcn4 may act through an intermediate TF Gln3 since both Gat1 and Gcn4 can separately form a protein complex with Gln3 [25].

In the third case study, we would like use a tool in YCRD to identify important CTFPs which regulate a set of ~800 cell cycle genes retrieved from Spellman et al.’s study [31]. So we (i) input a set of ~800 cell cycle genes, (ii) choose TFB&TFR as the experimental evidence of the regulatory association, and (iii) set 10^−12^ as the threshold of the Bonferroni-corrected p-value (see Fig 4A). YCRD then returns four important CTFPs (Mbp1-Swi4, Swi4-Tec1, Mcm1-Ste12 and Swi4-Swi6) which regulate the input set of genes (see Fig 4B). We validate the biological relevance of the four identified CTFPs as follows. First, the two TFs of each CTFP have physical interaction [25], implying that they may cooperatively regulate target genes. Second, all the TFs in the four identified CTFPs are either well known (Mbp1, Mcm1, Swi4, Swi6) [32] or predicted (Ste12 and Tec1) cell cycle TFs [8,33–35], indicating that our tool can identify important cell cycle-related CTFPs which regulate a set of cell cycle genes.

## Conclusions

In this article, we present the YCRD which provides 434,197 experimentally validated regulatory associations between 2535 cooperative TF pairs and 6243 target genes. YCRD has an easy-to-use interface for biologists to retrieve the target genes of a cooperative TF pair of interest or the cooperative TF pairs which regulate a gene of interest. Moreover, YCRD provides a tool for identifying important cooperative TF pairs which regulate the given set of genes. This is a very useful tool because biologists often have a set of genes of interest (e.g. upregulated genes under a specific biological condition) and would like to know the important cooperative TF pairs which regulate this set of genes. By identifying important cooperative TF pairs, biologists can form hypotheses of the combinatorial regulation of gene expression and have candidates to do further experimental investigation. YCRD will be regularly updated based on the newly published CTFPs identification algorithms and the latest releases of the BioGRID and YEASTRACT databases. We believe that the experimentally validated regulatory associations between cooperative TF pairs and their target genes deposited in YCRD will be a very useful resource for yeast biologists to study combinatorial regulation of gene expression.

## Supporting Information

S1 File For each cooperative TF pair, the number of target genes validated by TFB evidence without/with the same experimental condition is provided. (XLSX)
